# Alteration of the serum levels of the epidermal growth factor receptor and its ligands in patients with non-small cell lung cancer and head and neck carcinoma

**DOI:** 10.1038/sj.bjc.6603770

**Published:** 2007-04-24

**Authors:** Y Lemos-González, F J Rodríguez-Berrocal, O J Cordero, C Gómez, M Páez de la Cadena

**Affiliations:** 1 Departamento de Bioquímica, Genética e Inmunología, Universidad de Vigo, As Lagoas-Marcosende s/n, 36310, Vigo, Spain; 2 Departamento de Bioquímica y Biología Molecular, Universidad de Santiago de Compostela, San Francisco s/n, 15782, Santiago de Compostela, Spain; 3 Servicio de Oncología Médica, Complejo Hospitalario Universitario de Vigo, Pizarro 22, 36204, Vigo, Spain

**Keywords:** epidermal growth factor receptor, epidermal growth factor, transforming growth factor-alpha, amphiregulin, non-small cell lung cancer, head and neck carcinoma

## Abstract

Serum levels of the soluble epidermal growth factor receptor (sEGFR) and its ligands epidermal growth factor (EGF), transforming growth factor-*α* (TGF-*α*) and amphiregulin (AR) were measured in healthy donors and patients with non-small cell lung cancer (NSCLC) and head and neck carcinoma (HNC). In NSCLC, we found sEGFR and EGF levels significantly lowered in patients with respect to healthy donors. In HNC patients, significantly diminished levels were found in the case of sEGFR, EGF and also AR. In both malignancies, no significant association was found between the serum levels of the molecules and the patients' gender, age or smoking habit. Only a significant association was found between the decrease of sEGFR and the absence of distant metastasis in NSCLC and the tumour stage in HNC. The most interesting result was that combining sEGFR and EGF, sensitivities of 88% in NSCLC and 100% in HNC were reached without losing specificity (97.8% in both cases). The use of discriminant analysis and logistic regression improved the sensitivity for NSCLC and the specificity for HNC. These data demonstrate a potentially interesting value of the serum levels of sEGFR and EGF, especially when combined, as markers for NSCLC and HNC.

The epidermal growth factor receptor (EGFR, also known as HER1 or ErbB1) is a member of the HER family of membrane receptors. It is expressed on the surface of epithelial cells and presents an intracellular tyrosine kinase (TK) activity and one extracellular domain (termed as soluble epidermal growth factor receptor (sEGFR)) that can be shed and released into the bloodstream (Baron *et al*, 2003). The specific ligands for the EGFR are the epidermal growth factor (EGF), transforming growth factor-*α* (TGF-*α*) and amphiregulin (AR), although it can also bind betacellulin and epiregulin, which are also ligands for HER4 (Casalini *et al*, 2004).

Analysis of tumour levels of the EGFR as assayed by immunohistochemistry or in tumour lysates has shown that its overexpression is correlated with disease progression, poor survival and development of resistance to cytotoxic agents, being involved in over 70% of all cancers (Baselga, 2002; Jimeno and Hidalgo, 2005), specially in non-small cell lung cancer (NSCLC) and head and neck carcinoma (HNC). Recently, tumours that overexpress the EGFR have been challenged with the anti-EGFR therapies currently approved (Jimeno and Hidalgo, 2005; Mendelsohn and Baselga, 2006).

In addition to the traditional tissue-based analysis, there is an evident interest on the evaluation of the serum levels of the EGFR and its ligands, not only because they could indicate the presence of the malignancy but for their potential utility in the follow-up of patients, especially those receiving anti-EGFR therapies. Unfortunately, the number of publications that have analysed the levels of the receptor in human serum or plasma samples is rather small. Some of these studies dealt with squamous cell cancer of the head and neck (Hoffmann *et al*, 2001; Gokhale *et al*, 2005), thymoma (Sasaki *et al*, 2004), ovarian (Baron *et al*, 2003) and lung cancer (Sasaki *et al*, 2003), but comparison of the published data reveals a great disparity. On the other hand, studies evaluating the levels of the ligands in serum are very scarce and we have not found any study evaluating the levels of EGFR and their ligands in the same set of individuals.

Thus, considering this study as a first step to further explore the utility of these molecules as tumour markers, we aimed to determine the levels of sEGFR and their ligands in the serum of healthy donors and compare them with the levels in patients of NSCLC and HNC. We also evaluated if there was any correlation between the serum levels of the receptor and the cytokines and the clinicopathological features of the patients. Our data show that the levels of serum sEGFR, EGF, TGF-*α* and AR are altered in patients of both NSCLC and HNC. Moreover, these serum levels could have a potential clinical value in the detection/management of both diseases.

## SUBJECTS AND METHODS

### Subjects

Serum samples from 25 patients with NSCLC and 50 patients with HNC were collected at the hospitals ‘CHUVI’ (Complexo Hospitalario Universitario de Vigo) and ‘POVISA’ in Vigo (Spain). Hospital records and pathology slides were revised and TNM staging was made according to the 2002 UICC classification (Sobin and Wittekind, 2002). Informed consent was obtained from participants before entering the study and only cancer patients with untreated disease were included. The mean age (±s.d.) of the patients was 62±14 years (median: 61; range: 37–82) for NSCLC and 61±10 years (median: 60; range: 42–91) for HNC. Sera from 51 healthy donors were provided by ‘Centro de Transfusión de Galicia’ (Spain). The number of samples evaluated for each of the molecules included in this study is shown in the Supplementary Table 1.

### Determination of serum sEGFR, EGF, TGF-*α* and AR levels

Serum samples from controls and patients were obtained by venipuncture and blood clotting as described before (Cordero *et al*, 2000) and assayed by ELISA tests. Serum levels of sEGFR were measured with the EGFR Duoset® kit (DY231; R&D Systems, Minneapolis, MN, USA); EGF levels with the Quantikine® EGF kit (DEG00, R&D Systems); TGF-*α* levels with the Quantikine® TGF-*α* kit (DTGA00, R&D Systems) and AR levels with the AR Duoset® kit, (DY262, R&D Systems). For all the evaluated molecules, the method was based on the addition of diluted serum samples and standards to microtiter wells (Costar, Corning, NY, USA), precoated with a specific capture antibody. After incubation at room temperature, a conjugated detector antibody and a chromogenic solution were added and the reaction was stopped. Colorimetric quantification was performed with a microplate reader (550; Bio-Rad, Hercules, CA, USA) using dual readings at 450/570 nm. Protein concentrations were determined from the standard curves obtained according to the manufacturer's protocol.

The reproducibility of each commercial kit employed in this work was found to be coincident with the values provided by the manufacturer.

### Statistical methods

All the statistical tests were performed with the SPSS software package (release 14.0). The normal distribution of the variables was assessed by the non-parametric Kolmogorov–Smirnov test and the homogeneity of variances was evaluated using Levene's test. Although many of the variables studied presented a normal distribution and homogeneous variances, non-parametric tests were used since the number of samples tested was lower than 30 individuals for some of the groups analysed (see Supplementary Table 1). Correlations between variables were assessed by Spearman's rank-correlation coefficient and differences between groups compared with the Mann–Whitney's *U* test. An abnormal elevation of the serum levels of the molecules was defined as any value above the mean value for the healthy group plus two standard deviations and an abnormal reduction as any value below the mean value for the control group minus two standard deviations. These abnormal values were considered as positive for the marker in consideration. Receiver operating characteristics curves (ROCs) were constructed as plots of the percentage of true-positives (sensitivity) against the percentage of false-positives (100-specificity), to calculate the area under each curve (Cordero *et al*, 2000). Differences in positivity were compared with the clinicopathologic characteristics using the analysis of variance (Ayude *et al*, 2003–2004). All the statistical tests were two-sided and a *P*-value smaller than 0.05 was considered as statistically significant.

To increase the information obtained from the four molecules tested (sEGFR, EGF, TGF-*α* and AR), the sensitivity values previously calculated for each of them were combined, considering those values as the number of true-positives in the test. Similarly, specificity values were used as the number of false-positives detected by the molecules.

We also applied the discriminant analysis (DA), which attempts to find one or more linear combinations of independent variables to get the better separation of different groups of cases, to evaluate the utility of the different combinations of the four molecules for the discrimination between donors and patients. Besides, we tried the logistic regression (LR) where each marker is also included as a linear term in an equation. Once probabilities of having cancer were estimated, by DA and LR, we generated ROC curves to predict the existence of cancer from the positivity of multiple markers.

## RESULTS

### Serum levels of sEGFR and its ligands in healthy and NSCLC patients

Serum levels of sEGFR, EGF, TGF-*α* and AR in NSCLC patients were compared with the levels in healthy donors and results are shown in Table 1. In the healthy population, used as control group, the serum levels of sEGFR, EGF, TGF-*α* and AR were found to fit a normal distribution. The mean level of sEGFR was 35.9±5.2 ng ml^−1^. Levels of the specific EGFR ligands were lower than those obtained for the receptor: the mean value for the circulating EGF was 917.4±245.9 pg ml^−1^, whereas TGF-*α* showed an average of 6.0±4.2 pg ml^−1^ and AR presented a mean value of 19.6±17.4 pg ml^−1^. The levels of the four molecules were not correlated in donors as found by the non-parametric Spearman's rank-correlation coefficient.

**Figure 1:**
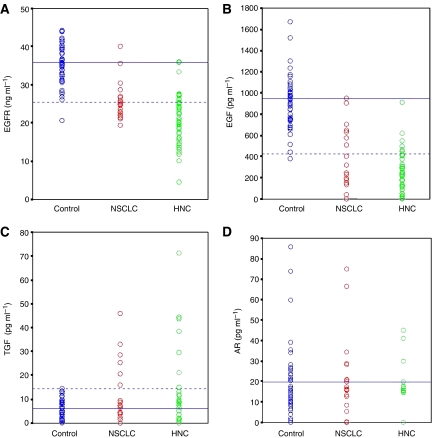
Representation of (**A**) sEGFR levels (ng ml^−1^) in sera from 50 healthy donors, 25 patients with NSCLC and 50 HNC patients; (**B**) EGF levels (pg ml^−1^) in sera from 45 healthy donors, 25 patients with NSCLC and 41 HNC patients; (**C**) TGF-*α* levels (pg ml^−1^) in sera from 44 healthy donors, 25 patients with NSCLC and 34 HNC patients; (**D**) AR levels (pg ml^−1^) in sera from 45 healthy donors, 24 patients with NSCLC and 25 HNC patients. Values of sEGFR and its ligands followed a normal distribution. The continuous blue line represents the mean of the control group, whereas the dashed blue line shows the lower normal limit (mean−2s.d. of the control group) for sEGFR, EGF and AR and the upper normal limit (mean+2s.d. of the control group) for TGF-*α*.

When the serum levels of sEGFR were analysed in NSCLC patients (Table 1), we found a mean of 25.5±4.5 ng ml^−1^. These levels were significantly decreased (*P*<0.0001) when compared with those of the control group (Figure 1A). Considering a cutoff value of 25.5 ng ml^−1^ (mean value of the healthy group −2s.d.) for sEGFR positivity, decreased serum sEGFR levels were observed in 15 out of 25 NSCLC patients and in one out of 50 donors, representing a 60% sensitivity with a 98% specificity. The area under the ROC curve (data not shown) was 0.925 (*P*<0.0001).

In the case of serum EGF (Figure 1B and Table 1), its average levels were 294.3±298.2 pg ml^−1^, significantly lower than those of controls (*P*<0.0001) although they presented a higher dispersion of values. Decreased EGF was found in 72% (18 out of 25) of the patients with a 97.8% specificity (1 out of 45 donors was detected as positive for cancer), when the mean value of the healthy group minus two standard deviations (425.6 pg ml^−1^) was used as the cutoff point. The area under the ROC curve (data not shown) was 0.940 (*P*<0.0001), higher than the one obtained for sEGFR.

For TGF-*α* (Figure 1C and Table 1), though not statistically significant, the mean levels (9.2±12.3 pg ml^−1^) were higher in patients than in controls. However, the median, (4.3 pg ml^−1^, range: 0–45.9) was lower because of the number of non-detected (ND) values (Supplementary Figure 1). For a cutoff value of 14.4 pg ml^−1^ (mean of the healthy group plus two standard deviations), positivity was observed in six out of 25 NSCLC patients (20.0%), with a 97.7% specificity (one out of 44 donors).

Regarding AR (Figure 1D and Table 1), an average of 17.2±19.4 pg ml^−1^ was found, not showing significant differences with respect to control values. In this case, we considered the lower limit of AR levels (0.0 pg ml^−1^) as a cutoff point value. ND values were observed in six out of 24 NSCLC patients (25.0%) with a 95.6% specificity (two out of 45 donors).

The levels of the molecules studied did not show statistical correlation in NSCLC patients.

### Relationship between the serum levels of sEGFR and its ligands, and the clinicopathological features in NSCLC patients

No significant association was found between the serum protein levels of each of the molecules evaluated and patients' gender, age, smoking habits or the tumour pathological subtype and stage (Tables 2 and 3). Only the absence of distant metastasis was significantly (*P*=0.040) associated with decreased levels of sEGFR (Table 2) and 100% of the patients without distant metastasis were positive for the sEGFR.

### Combined use of sEGFR, EGF, TGF-*α* and AR serum levels for NSCLC detection

The lack of correlation among the molecules analysed prompted us to evaluate their performance when combining their cutoff points to increase the sensitivity and/or the specificity of the test. Thirty-eight donors and 24 NSCLC patients were included in this analysis since their samples had been evaluated for the four molecules. In Supplementary Table 2, we show these parameters for all the combinations of measurements. Using the cutoff points mentioned before, the combined use of sEGFR and EGF levels rendered the best results, since sensitivity was increased to 88% maintaining a high specificity (97.8%).

**Figure 2:**
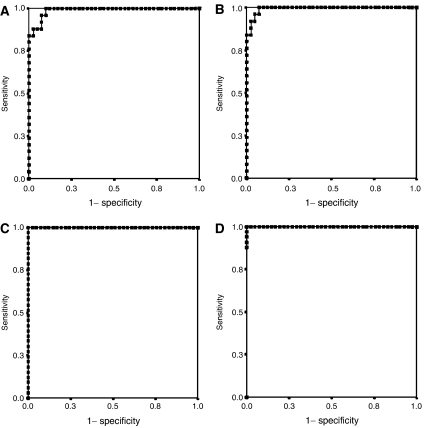
ROCs for the combined levels of sEGFR, EGF and TGF-*α* when applied to the comparison of healthy donors and NSCLC patients by DA (**A**) and LR (**B**) or to the comparison of donors and HNC patients by DA (**C**) and LR (**D**).

An alternative method to distinguish between donors and patients is to use the DA. The best results were obtained combining sEGFR, EGF and TGF-*α* levels, which allowed the correct classification of 90.2% of the healthy individuals and 100% of the NSCLC patients. A ROC curve was constructed for the combination of those three molecules, showing an area under the curve of 0.989 (Figure 2A). Interestingly, the combination of sEGFR and EGF levels presented almost the same efficacy, with an area under the corresponding ROC curve of 0.982 (data not shown). The LR model using those three markers also yielded a wide area under the ROC curve, with a value of 0.993 (Figure 2B), correctly classifying 92.7% of the donors and 96% of the patients.

### Serum levels of sEGFR and its ligands in HNC patients

Serum levels of sEGFR, EGF, TGF-*α* and AR in HNC patients were compared with the levels in healthy donors, the results obtained are shown in Table 4. Analysis of the sEGFR levels in HNC patients showed a mean value of 21.2±6.2 ng ml^−1^. When these values were compared with those obtained for the donor group, we found significant differences (*P*<0.0001) that were also revealed for the comparison with the NSCLC group (*P*=0.002) (Figure 1A). Using 25.5 ng ml^−1^ as the cutoff value (set before for the sEGFR levels as the mean–2s.d. in donors) positivity for the sEGFR was observed in 40 out of the 50 HNC patients (80% sensitivity with 98% specificity). The approximate area under the ROC curve (data not shown) was 0.961 (*P*<0.0001).

EGF levels in HNC patients showed a mean of 230.3±204.2 pg ml^−1^ (Table 4), also significantly lower (*P*<0.0001) than those of controls (Figure 1B). Selecting a cutoff value of 425.6 pg ml^−1^ (mean−2s.d. in donors) positivity of EGF was found in 82.9% (34 out of 41) of the patients (specificity of 97.8%). The area under the ROC curve built for EGF (data not shown) was 0.979 (*P*<0.0001).

The mean TGF-*α* value in HNC was 20.5±48.6 pg ml^−1^ (Table 4 and Figure 1C). As in NSCLC, a trend to increased TGF-*α* levels was observed in HNC (Supplementary Figure 2) and nine out of 34 patients were positive (23.5% sensitivity with 97.7% specificity) when setting a cutoff point of 14.4 pg ml^−1^ (mean+2s.d. in donors). Finally, the mean AR value was 10.5±13.3 pg ml^−1^ (Table 4 and Figure 1D). In contrast with NSCLC, the difference between donors and patients was statistically significant, defining a cutoff value of 0.0 pg ml^−1^, AR was undetectable and therefore positive for the test in 13 out of 25 HNC patients (52% sensitivity with a 95.6% specificity). The area under the ROC curve for AR levels (data not shown) was significant (*P*=0.004), with a value of 0.706.

A weak but significant negative correlation between sEGFR and AR, neither detected in NSCLC nor in donors, was demonstrated for the HNC group (*ρ*=−0.419).

### Relationship between the serum levels of sEGFR and its ligands, and the clinicopathological features in HNC patients

The study of the relationship between the serum levels of sEGFR and EGF (Table 5) and TGF-*α* and AR (Table 6) and the clinicopathological features of the patients demonstrated a significant association between the decrease of sEGFR levels and the tumour stage (*P*=0.011). We also found an association between decreased levels of sEGFR and the patients age that, although significant (*P*=0.005), could be related to the fact that advanced stages correlated with older patients (data not shown).

### Combined use of sEGFR EGF, TGF-*α* and AR serum levels for HNC detection

As shown before, the serum levels of sEGFR or EGF alone are acceptably informative about the existence of HNC. Nevertheless, we tried the combination of the four molecules tested to enhance the sensitivity of the tests (Supplementary Table 3). Interestingly, just with the combination of sEGFR and EGF levels, we detected all the 40 HNC patients (100% sensitivity) with 97.8% specificity. In addition, the statistical tests applied with the same three molecules used for NSCLC (sEGFR, EGF and TGF-*α*) allowed a correct discrimination of 97.6% of the healthy donors and 100% of the HNC patients by DA and 100% of the healthy donors and 100% of the HNC patients by LR. In fact, a wide area under the ROC curve, with a value of 1.000 (*P*<0.0001), was obtained by both DA and LR (Figure 2C and D), although just with the combination of sEGFR and EGF the area under the ROC curve was 0.999 (*P*<0.0001) (data not shown).

## DISCUSSION

Novel serum markers are still needed in the case of malignancies such as NSCLC and HNC, not only for diagnosis but also for prognosis evaluation and follow-up of the patients. The EGFR is a strong biomarker candidate for multiple reasons. First of all, it is overexpressed in most of the tumours from NSCLC and HNC patients. This overexpression, ultimately causing increased proliferation or cell motility, and decreased apoptosis (Jimeno and Hidalgo, 2005), has been related to the progression of the tumour. Another appealing reason is the fact that new approved therapies for those cancers are targeting EGFR, based on the inhibition of its TK activity (Baselga, 2002; Jimeno and Hidalgo, 2005).

On the other hand, it is known that a portion of the membrane receptor is shed into the bloodstream thus making plasma or serum an excellent source, best than tumour tissues, to evaluate dynamic variations of the EGFR since the diagnosis and during treatment.

Moreover, it has been described that overexpression of the receptor is often associated with an increased expression of its ligands (Rubin Grandis *et al*, 1998). This and the evidence that the combined use of several markers can improve the sensitivity and specificity of the test (Ayude *et al*, 2003–2004; Lu *et al*, 2004; Skates *et al*, 2004) prompted us to measure the levels of sEGFR EGF, TGF-*α* and AR in the serum of healthy individuals and patients with NSCLC or HNC. This work aimed first to establish an average value for the control population, and then to study the variations in patients, correlating the levels of the four molecules with the classical clinicopathological conditions. Finally, to explore the putative role of these proteins as tumour biomarkers, we combined the values of the four molecules applying multivariate analyses.

To our knowledge this is the first study where the serum levels of EGFR and its ligands are measured in the same set of healthy individuals, NSCLC patients and HNC patients. Previous reports about the levels of sEGFR in healthy individuals showed discrepant values, ranging from 4.5 to 159.1 ng ml^−1^ (Choi *et al*, 1997; Hoffmann *et al*, 2001; Markopoulos *et al*, 2001; Ramesh Kumar *et al*, 2001; Carney *et al*, 2002; Baron *et al*, 2003; Kong *et al*, 2004; Gokhale *et al*, 2005). Even using the same commercial ELISA kit, the differences are remarkable: 36.4 ng ml^−1^, in our study, *vs* 74.8 ng ml^−1^ reported by Baron *et al* (2001). EGF levels also presented a wide range of variation in donors, from 71.9 to 490.5 pg ml^−1^ (Baron *et al*, 1999; Efimova *et al*, 2005; Hashimoto *et al*, 2005), all these values lower than the ones reported here. Studies on TGF-*α* levels in healthy subjects reported ranges from non-detectable levels to 56.2 pg ml^−1^ (Tomiya and Fujiwara, 1996; Chien *et al*, 1997; Shim *et al*, 1998; Choi *et al*, 1999; Harada *et al*, 1999; Efimova *et al*, 2005). On the other hand, there are no reports on AR in serum of healthy populations. Therefore, comparison between different populations should be carefully addressed.

In this study, sEGFR and EGF levels were significantly decreased in serum of NSCLC and HNC patients, whereas there was no difference in TGF-*α* and AR levels were significantly lower only in HNC patients. Studies reported to date about sEGFR levels in NSCLC are controversial. For instance, Schneider *et al* (1999) did not find any alteration of sEGFR levels in occupation-derived lung cancer patients. However, decreased serum sEGFR has been reported in 42% of lung cancer patients by other authors (Carney *et al*, 2002). As those studies included patients with heterogeneous types of lung cancer, it is difficult to compare them with the one reported here, since only non-small cell cancer patients were included. On the other hand, two studies on HNC, using different commercial kits, have reported no differences in sEGFR serum levels in squamous cell carcinoma (Hoffmann *et al*, 2001; Gokhale *et al*, 2005), although our results demonstrated that sEGFR could detect HNC patients with an 80% sensitivity. A decrease in sEGFR serum levels has also been shown in breast, ovarian, colon, bladder and prostate carcinoma (Carney *et al*, 2002). On the contrary, in cervical (Oh *et al*, 2000), gastric (Choi *et al*, 1997) and pituitary carcinomas (Kong *et al*, 2004), a significant increase of sEGFR has been detected.

This is the first report on circulating EGF, measured by ELISA in NSCLC and HNC. EGF concentrations were found lowered in both malignancies, in agreement with previous data in serum of thyroid carcinoma patients (Nedvídková *et al*, 1992). However, an increase of EGF was found in pancreatic (Meggiato *et al*, 1999) and papillary thyroid carcinoma (Konturek *et al*, 2005), and no alterations were found in ovarian cancer (Baron *et al*, 1999), suggesting that its biological function is tissue-dependent.

TGF-*α* levels did not change significantly in NSCLC and HNC but showed a trend to higher levels, consequent with reports confirming increased serum TGF-*α* in ovarian (Chien *et al*, 1997), hepatocellular (Tomiya and Fujiwara, 1996; Harada *et al*, 1999), colorectal (Shim *et al*, 1998), gastric (Moskal *et al*, 1995; Choi *et al*, 1999) and breast cancer (Chakrabarty *et al*, 1994) as measured by RIA. For AR, we found a significant decrease in HNC, whereas in NSCLC patients only a trend to impairment was detected. To our knowledge, there is only one report on serum AR and TGF-*α* in advanced NSCLC (healthy donors not measured) studying the relationship of their serum levels with the response of patients to gefitinib (Ishikawa *et al*, 2005). In that work, AR and TGF-*α* levels were detected with the same commercial antibodies used here and were quite similar to the ones shown by us. Our data also agree with their observation of a high-value dispersion. Interestingly, those authors proposed that the value of AR correlates with the distinct response of the patients to the drug (Ishikawa *et al*, 2005). Therefore, further studies to establish if the serum levels of AR during treatment are useful for predicting chemosensitivity of patients, will be of great aid.

Interestingly, sEGFR levels in stage I HNC tumours are a great deal lower than those in the healthy population, so as to support further studies regarding early diagnosis (either alone or in combination with other molecules). Because of the degree of sample's positivity and the dramatically altered levels, the same comment applies to EGF. Unfortunately, in the case of NSCLC, we could not confirm the correlation between early stages and lower levels of sEGFR since in this type of cancer patients are usually diagnosed in advanced stages.

For many cancers, up to date there is not a single diagnostic test able to detect early-stage tumours. This also applies to prognosis in spite of the easier management of tumours or biopsies in this case. However, the number of potential molecular markers is constantly increasing. For serum biomarkers, multivariate analysis is not usually feasible because of the analysis of a single molecule at a time. However, it has been recently demonstrated that combining data from several markers by multivariate methods clearly improves the sensitivity and the specificity of the tests (Lu *et al*, 2004; Skates *et al*, 2004). Here, we studied four putative markers in the same sera that allowed a multivariate analysis to detect the independence of those factors in relation to cancer diagnosis. An interesting result from our data is that combining sEGFR and EGF, sensitivities of 88% in NSCLC and 100% in HNC are reached without losing specificity (97.8% in both cases). The use of statistical methods, such as DA and LR improved the sensitivity for NSCLC. Thus, applying DA combining sEGFR, EGF and TGF-*α*, 90.2% of the healthy individuals and 100% of the NSCLC patients were correctly classified. Regarding HNC, LR, on the combination of sEGFR, EGF and TGF-*α*, correctly classified 100% of the healthy donors and 100% of the HNC patients.

In conclusion, in this study we present a number of results indicating the potential clinical utility of sEGFR, EGF, TGF-*α* and AR in the management of NSCLC and HNC diseases. Further studies are of interest to evaluate their use in diagnosis, prognosis and disease therapy monitoring.

## Figures and Tables

**Table 1:** Serum levels of sEGFR, EGF, TGF-*α* and AR in NSCLC patients compared with levels in healthy donors

	**Serum**	***n***	**M±s.d.**	**Me**	**Range**	**Mann–Whitney *U*-test**
sEGFR						
(ng ml^−1^)	Donors	50	35.9±5.2	36.4	20.6–44.2	
	NSCLC	25	25.5±4.5	25.3	19.4–40.1	*P*<0.0001
						
EGF						
(pg ml^−1^)	Donors	45	917.4±245.9	946.7	382.3–1671.0	
	NSCLC	25	294.3±298.2	196.6	0.0–952.4	*P*<0.0001
						
TGF-*α*						
(pg ml^−1^)	Donors	44	6.0±4.2	6.2	0.0–14.5	
	NSCLC	25	9.2±12.3	4.3	0.0–45.9	NS
						
AR						
(pg ml^−1^)	Donors	45	19.6±17.4	15.4	0.0–85.8	
	NSCLC	24	17.2±19.4	14.6	0.0–74.8	NS

AR=amphiregulin; EGF=epidermal growth factor; NSCLC=non-small cell lung cancer; sEGFR=soluble epidermal growth factor receptor; TGF=transforming growth factor; M±s.d.=mean±standard deviation; Me=median; n=number of samples; NS=nonsignificant.

**Table 2:** Serum values of sEGFR and EGF in NSCLC patients according to the clinicopathological features

**Characteristics**	**sEGFR mean (ng ml^−1^)**	**ANOVA *P***	**Positive cases**	**EGF mean (pg ml^−1^)**	**ANOVA *P***	**Positive cases**
*Gender*		0.639			0.568	
Female	24.5		3/5 (60.0%)	398.3		3/5 (60.0%)
Male	25.5		10/17 (58.8%)	307.8		12/17 (70.6%)
						
*Age*		0.108			0.695	
⩽60	26.7		4/11 (36.4%)	354.5		7/11 (63.6%)
>60	23.8		9/11 (81.8%)	302.2		8/11 (72.7%)
						
*Smoking habits*		0.746			0.786	
Non-smoker	24.1		2/4 (50.0%)	417.6		3/4 (75.0%)
Smoker	25.7		10/17 (58.8%)	303.2		11/17 (64.7%)
Ex-smoker	23.6		1/1 (100%)	400.0		1/1 (100%)
						
*Pathological subtype*		0.278			0.129	
Adenocarcinoma	24.7		4/7 (57.1%)	300.6		5/7 (71.4%)
Epidermic	23.0		5/6 (83.3%)	237.2		5/6 (83.3%)
Large cell	21.1		1/1 (100%)	952.4		0/1 (0.0%)
Unclassified NSCLC	28.1		3/8 (37.5%)	343.1		5/8 (62.5%)
						
*Distant metastasis*		0.040			0.702	
M0	21.5		4/4 (100%)	382.5		3/4 (75.0%)
M1	26.1		9/18 (50.0%)	316.3		12/18 (66.7%)
						
*Stage*		0.128			0.779	
IIIa	21.5		2/2 (100%)	476.2		1/2 (50.0%)
IIIb	21.5		2/2 (100%)	288.8		2/2 (100%)
IV	26.1		9/18 (50.0%)	316.3		12/18 (66.7%)

ANOVA=analysis of variance; EGF=epidermal growth factor; NSCLC=non-small cell lung cancer; sEGFR=soluble epidermal growth factor receptor.

*P-*values were calculated using one-way ANOVA. *P-*values <0.05 were considered significant.

Positivity was defined as any value below the mean value of the donor group−2s.d.

The pathological subtype named as ‘Unclassified NSCLC’ was not included in the statistical analysis.

For each condition, the significance of the values was studied within the patient cohort.

**Table 3:** Serum values of the TGF-*α* and the AR in NSCLC according to the clinicopathological features

**Characteristics**	**TGF-*α* mean (pg ml^−1^)**	**ANOVA *P***	**Positive cases**	**AR mean (pg ml^−1^)**	**ANOVA *P***	**Positive cases**
*Gender*		0.281			0.783	
Female	14.6		2/5 (40.0%)	20.4		2/5 (40.0%)
Male	7.6		3/17 (17.6%)	17.5		3/17 (17.6%)
						
*Age*		0.382			0.630	
⩽60	11.6		3/11 (27.3%)	20.3		2/11 (18.2%)
>60	6.8		2/11 (18.2%)	16.0		3/11 (27.3%)
						
*Smoking habits*		0.709			0.313	
Non-smoker	11.9		2/4 (50.0%)	30.2		1/4 (25.0%)
Smoker	9.1		3/17 (17.6%)	16.4		3/17 (17.6%)
Ex-smoker	0.0		0/1 (0.0%)	0.0		1/1 (100%)
						
*Pathological subtype*		0.402			0.452	
Adenocarcinoma	11.8		2/7 (28.6%)	27.1		2/7 (28.6%)
Epidermic	3.4		0/6 (0.0%)	10.4		1/6 (16.7%)
Large cell	0.0		0/1 (0.0%)	13.3		0/1 (0.0%)
Unclassified NSCLC	12.4		3/8 (37.5%)	16.7		2/8 (25.0%)
						
*Distant metastasis*		0.271			0.242	
M0	2.9		0/4 (0.0%)	7.4		1/4 (25.0%)
M1	10.6		5/18 (27.8%)	20.5		4/18 (22.2%)
						
*Stage*		0.550			0.513	
IIIa	2.2		0/2 (0.0%)	6.8		0/2 (0.0%)
IIIb	3.7		0/2 (0.0%)	8.0		1/2 (50.0%)
IV	10.6		5/18 (27.8%)	20.5		4/18 (22.2%)
						

ANOVA=analysis of variance; AR=amphiregulin; NSCLC=non-small cell lung cancer; TGF=transforming growth factor.

*P-*values were calculated using one-way ANOVA. *P*-values <0.05 were considered significant.

Positivity was defined as any value above (for TGF-*α*) or below (for AR) the mean value of the donor group+(for TGF-*α*) or −(for AR) 2s.d.

The pathological subtype named as “Unclassified NSCLC” was not included in the statistical analysis.

For each condition, the significance of the values was studied within the patient cohort.

**Table 4:** Serum levels of sEGFR, EGF, TGF-*α* and AR in HNC patients compared with levels in healthy donors

	**Serum**	***n***	**M±s.d.**	**Me**	**Range**	**Mann–Whitney *U*-test**
sEGFR (ng ml^−1^)	Donors	50	35.9±5.2	36.4	20.6–44.2	
	HNC	50	21.2±6.2	20.5	4.5–36.0	*P*<0.0001
						
						
EGF (pg ml^−1^)	Donors	45	917.4±245.9	946.7	382.3–1671.0	
	HNC	41	230.3±204.2	211.9	0.0–911.3	*P*<0.0001
						
						
TGF-*α* (pg ml^−1^)	Donors	44	6.0±4.2	6.2	0.0–14.5	
	HNC	34	20.5±48.6	8.4	0.0 – 280.0	NS
						
						
AR (pg ml^−1^)	Donors	45	19.6±17.4	15.4	0.0–85.8	
	HNC	25	10.5±13.3	0.0	0.0–45.0	*P*=0.008

AR=amphiregulin; EGF=epidermal growth factor; HNC=head and neck carcinoma; sEGFR=soluble epidermal growth factor receptor; TGF=transforming growth factor; *n*=number of samples; M±s.d.=mean±standard deviation; Me=median; NS=nonsignificant.

**Table 5:** Serum values of the sEGFR and the EGF in HNC according to the clinicopathological features

**Characteristics**	**sEGFR mean (ng ml^−1^)**	**ANOVA *P***	**Positive cases**	**EGF mean (pg ml^−1^)**	**ANOVA *P***	**Positive cases**
*Gender*		—			—	
Female	—		—	—		—
Male	21.2		40/50 (80.0%)	230.3		34/41 (82.9%)
						
*Age*		0.005			0.672	
⩽60	23.5		19/26 (73.1%)	217.6		19/22 (86.4%)
>60	18.7		21/24 (87.5%)	245.1		15/19 (78.9%)
						
*Smoking habits*		0.564			0.981	
Non-smoker	18.7		2/2 (100%)	235.1		1/1 (100%)
Smoker	21.3		38/48 (79.2%)	230.2		33/40 (82.5%)
						
*Tumor location*		0.613			0.404	
Larynx	21.7		20/26 (76.9%)	263.9		17/21 (81.0%)
Oropharynx	21.2		7/9 (77.8%)	196.7		8/10 (80.0%)
Hipopharynx	18.5		7/8 (87.5%)	233.5		4/5 (80.0%)
Oral cavity	22.4		5/5 (100%)	51.8		3/3 (100%)
						
*Tumor status*		0.081			0.201	
T0	27.6		0/1 (0.0%)	375.5		1/1 (100%)
T1	23.4		10/14 (71.4%)	315.1		9/13 (69.2%)
T2	23.2		8/12 (66.7%)	158.4		9/9 (100%)
T3	19.5		7/8 (87.5%)	106.3		6/6 (100%)
T4	18.1		14/14 (100%)	243.0		8/11 (72.7%)
Tx	16.2		1/1 (100%)	235.1		1/1 (100%)
						
*Nodal status*		0.231			0.799	
						
N0	22.9		18/25 (72.0%)	244.0		17/20 (85.0%)
N1	19.1		9/11 (81.8%)	174.5		6/7 (85.7%)
N2	20.0		11/12 (91.7%)	224.1		9/12 (75.0%)
N3	17.6		2/2 (100%)	326.2		2/2 (100%)
						
*Distant metastasis*		0.291			0.854	
M0	21.6		34/43 (79.1%)	227.6		29/34 (85.3%)
Mx	18.9		6/7 (85.7%)	243.5		5/7 (71.4%)
						
*Stage*		0.011			0.707	
I	24.5		7/11 (63.6%)	290.8		8/10 (80.0%)
II	24.7		2/4 (50.0%)	212.8		2/2 (100%)
III	22.7		9/12 (75.0%)	179.8		8/9 (88.9%)
IV	18.2		22/23 (95.7%)	224.6		16/20 (80.0%)
						
*Differentiation*		0.437			0.379	
Good	22.2		6/6 (100%)	270.6		3/4 (75.0%)
Moderate	21.8		24/33 (72.7%)	254.6		20/26 (76.9%)
Poor	19.0		9/10 (90.0%)	150.6		10/10 (100%)

ANOVA=analysis of variance; EGF=epidermal growth factor; sEGFR=soluble epidermal growth factor receptor; HNC=head and neck carcinoma.

*P*-values were calculated, and positivity was defined, as in Table 2.

Tumours that could not be clinically evaluated were classified as Tx and were not included in the statistical analysis.

For each condition, the significance of the values was studied within the patient cohort.

**Table 6:** Serum values of the TGF-*α* and the AR in HNC according to the clinicopathological features

**Characteristics**	**TGF-*α* mean (pg ml^−1^)**	**ANOVA *P***	**Positive cases**	**AR mean (pg ml^−1^)**	**ANOVA *P***	**Positive cases**
*Gender*		—			—	
Female	—		—	—		—
Male	20.5		9/34 (26.5%)	10.5		13/25 (52.0%)
						
*Age*		0.287			0.643	
⩽60	11.5		4/17 (23.5%)	9.1		7/12 (58.3%)
>60	29.5		5/17 (29.4)	11.7		6/13 (46.2)
						
*Smoking habits*		0.826			0.016	
Non-smoker	9.7		0/1 (0.0%)	41.0		0/1 (0.0%)
Smoker	20.8		9/33 (27.3%)	9.2		13/24 (54.2%)
						
*Tumor location*		0.360			0.089	
Larynx	15.4		4/16 (25.0%)	6.7		10/17 (58.8%)
Oropharynx	13.5		3/9 (33.3%)	20.5		1/3 (33.3%)
Hipopharynx	58.3		1/5 (20.0%)	22.2		0/2 (0.0%)
Oral cavity	8.1		1/2 (50.0%)	0.0		1/1 (100%)
						
*Tumor status*		0.639			0.122	
T0	11.3		0/1 (0.0%)	0.0		1/1 (100%)
T1	15.4		3/11 (27.3%)	14.0		3/7 (42.9%)
T2	12.9		3/8 (37.5%)	0.0		4/4 (100%)
T3	11.6		1/6 (16.7%)	20.3		0/3 (0.0%)
T4	47.8		2/7 (28.6%)	6.9		5/9 (55.6%)
Tx	9.7		0/1 (0.0%)	41.0		0/1 (0.0%)
						
*Nodal status*		0.581			0.115	
N0	13.2		4/15 (26.7%)	10.5		6/12 (50.0%)
N1	12.6		1/6 (16.7%)	9.0		3/5 (60.0%)
N2	37.6		4/11 (36.4%)	7.0		4/7 (57.1%)
N3	4.9		0/2 (0.0%)	41.0		0/1 (0.0%)
						
*Distant metastasis*		0.837			0.052	
M0	21.4		7/27 (25.9%)	8.6		13/22 (59.1%)
Mx	17.0		2/7 (28.6%)	24.3		0/3 (0.0%)
						
*Stage*		0.782			0.798	
I	18.2		3/8 (37.5%)	13.4		3/6 (50.0%)
II	3.6		0/2 (0.0%)	0.0		1/1 (100%)
III	9.8		1/8 (12.5%)	12.2		2/5 (40.0%)
IV	29.1		5/16 (31.3%)	9.2		7/13 (53.8%)
						
*Differentiation*		0.778			0.250	
Good	21.9		1/2 (50.0%)	0.0		4/4 (100%)
Moderate	24.6		7/23 (30.4%)	11.0		6/13 (46.2%)
Poor	9.8		1/8 (12.5%)	11.1		3/7 (42.9%)

ANOVA=analysis of variance; AR=amphiregulin; HNC=head and neck carcinoma; TGF=transforming growth factor.

*P-*values were calculated, and positivity was defined, as in Table 3.

Tumours that could not be clinically evaluated were classified as Tx and were not included in the statistical analysis.

For each condition, the significance of the values was studied within the patient cohort.
