# Supplementary material for: Alteration of the serum levels of the epidermal growth factor receptor and its ligands in patients with non-small cell lung cancer and head and neck carcinoma
Source: Br J Cancer. 2007 Apr 24;96(10):1569–78. doi: 10.1038/sj.bjc.6603770 (PMC2359945; doi:10.1038/sj.bjc.6603770)
Supplement: Supplementary Information [file 6603770x1.doc]

**SUPPLEMENTAL TABLES**

**Table 1:** Number of samples evaluated for each of the molecules included in the study.

| **Case** | **sEGFR** | **EGF** | **TGF-α** | **AR** |
| --- | --- | --- | --- | --- |
| Control | 50 | 45 | 44 | 45 |
| NSCLC | 25 | 25 | 25 | 24 |
| HNC | 50 | 41 | 34 | 25 |

**Table 2:** Diagnostic parameters for the detection of NSCLC as found for the serum levels of sEGFR or EGF alone or in combination with TGF-α and AR levels.

| **Variables** | **Sensitivity (%)** | **Specificity (%)** |
| --- | --- | --- |
| sEGFR | 60.0 | 98.0 |
| EGF | 72.0 | 97.8 |
| sEGFR + EGF | 88.0 | 97.8 |
| sEGFR + TGF-α | 68.0 | 97.7 |
| sEGFR + AR | 66.7 | 93.4 |
| EGF + TGF-α | 76.0 | 97.6 |
| EGF + AR | 87.5 | 95.1 |
| TGF-α + AR | 41.7 | 92.5 |
| sEGFR + EGF + TGF-α | 88.0 | 97.6 |
| sEGFR + EGF + AR | 91.7 | 92.5 |
| sEGFR + TGF-α + AR | 75.0 | 92.5 |
| EGF + TGF-α + AR | 83.4 | 92.3 |
| sEGFR + EGF + TGF-α + AR | 91.7 | 92.1 |

**Table 3:** Diagnostic parameters for the detection of HNC as found for the serum levels of sEGFR or EGF alone or in combination with TGF-α and AR levels.

| **Variables** | **Sensitivity (%)** | **Specificity (%)** |
| --- | --- | --- |
| sEGFR | 80.0 | 98.0 |
| EGF | 82.9 | 97.8 |
| sEGFR + EGF | 100 | 97.8 |
| sEGFR + TGF-α | 84.9 | 97.7 |
| sEGFR + AR | 92.0 | 93.4 |
| EGF + TGF-α | 91.2 | 97.6 |
| EGF + AR | 91.3 | 95.1 |
| TGF-α + AR | 43.8 | 92.5 |
| sEGFR + EGF + TGF-α | 94.0 | 97.6 |
| sEGFR + EGF + AR | 100 | 92.5 |
| sEGFR + TGF-α + AR | 87.5 | 92.5 |
| EGF + TGF-α + AR | 100 | 92.3 |
| sEGFR + EGF + TGF-α + AR | 100 | 92.1 |

**SUPPLEMENTAL FIGURES**

**Figure 1**


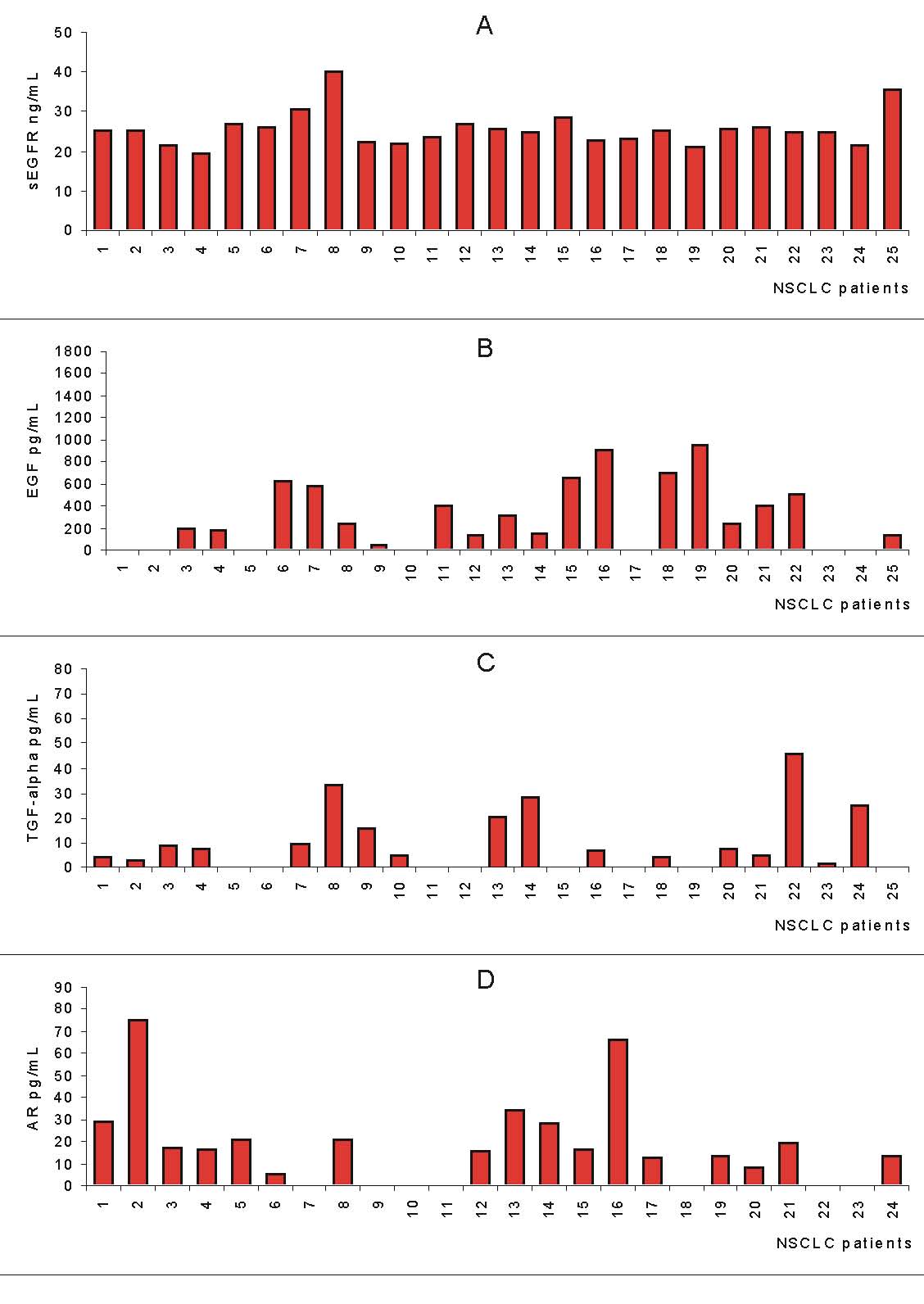


**SUPPLEMENTAL FIGURES**

**Figure 2**


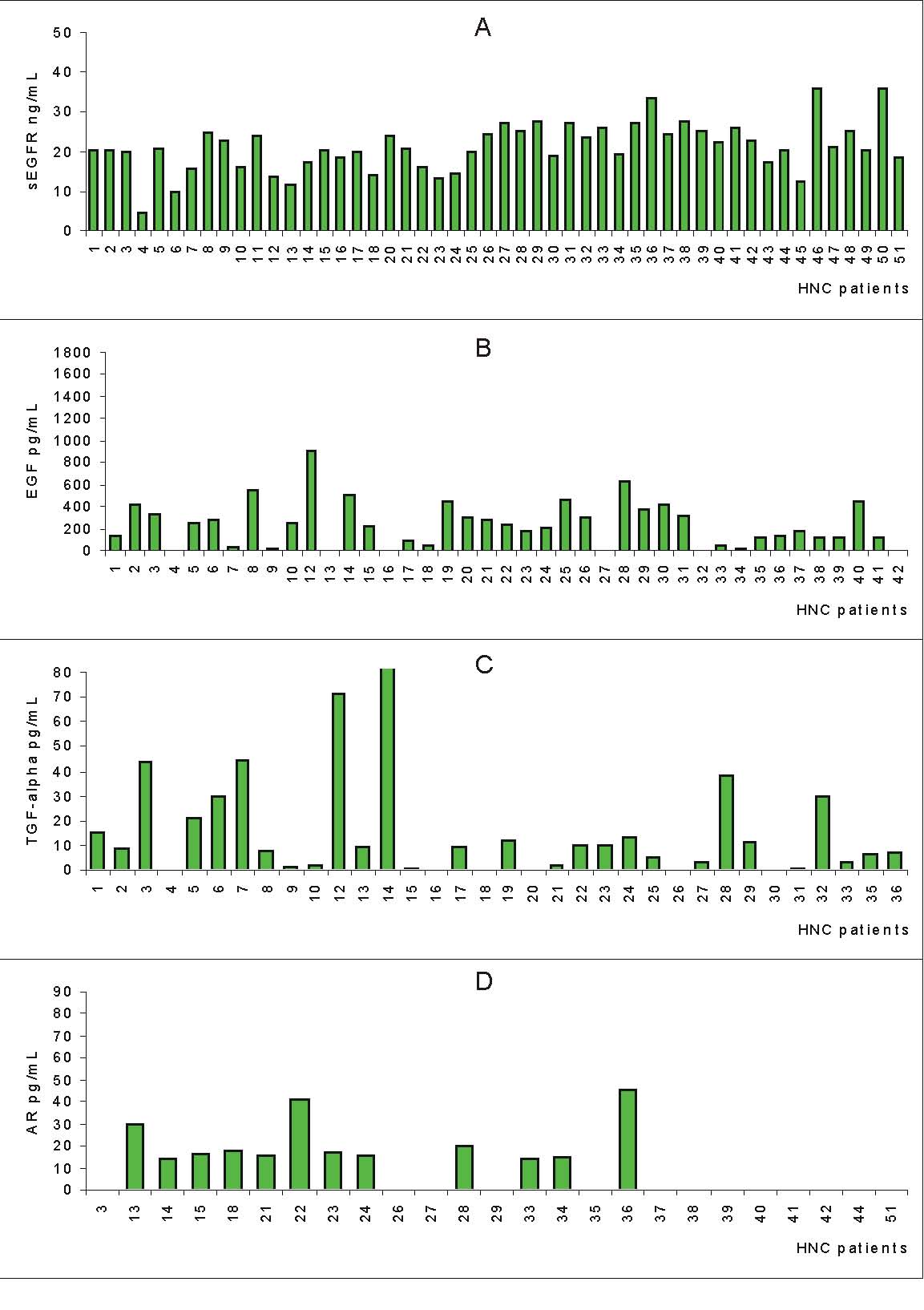


**LEGENDS OF THE SUPPLEMENTAL FIGURES**

**Figure 1:** Individual serum levels of (A) sEGFR (ng/mL), (B) EGF (pg/mL), (C) TGFα (pg/mL), (D) AR (pg/mL) in NSCLC patients.

**Figure 2:** Individual serum levels of (A) sEGFR (ng/mL), (B) EGF (pg/mL), (C) TGFα (pg/mL), (D) AR (pg/mL) in HNC patients.
